# Supplementary material for: Success of concrete and crab traps in facilitating Eastern oyster recruitment and reef development
Source: PeerJ. 2019 Feb 25;7:e6488. doi: 10.7717/peerj.6488 (PMC6394346; doi:10.7717/peerj.6488)
Supplement: Supplemental Information 5 — Detailed methods for mixing, molding and deploying the concrete structures used in our field experiment. [file peerj-07-6488-s005.pdf]

Supplement: Concrete structures specifications and construction

Description of concrete structures

Each concrete structure is composed of 12 modular blocks that interlock and stack in a 'Lincoln log' configuration. Two different block sizes provide a rectangular base with exterior dimensions of approximately 100 cm W x 50 cm L (Figs. S1 & S2). Block dimensions are approximately 50 cm W x 14 cm H x 9 cm W (large) and 50 cm W x 14 cm H x 9 cm W (small). Notches (9 cm W x 9 cm L x 4 cm H) near the ends of each block allow the blocks to interlock and stack without hardware. For each structure we stacked three blocks on each side, for a total height of approximately 42 cm. The blocks enclose an interior space whose basal dimensions are approximately 70 cm W x 25 cm L.

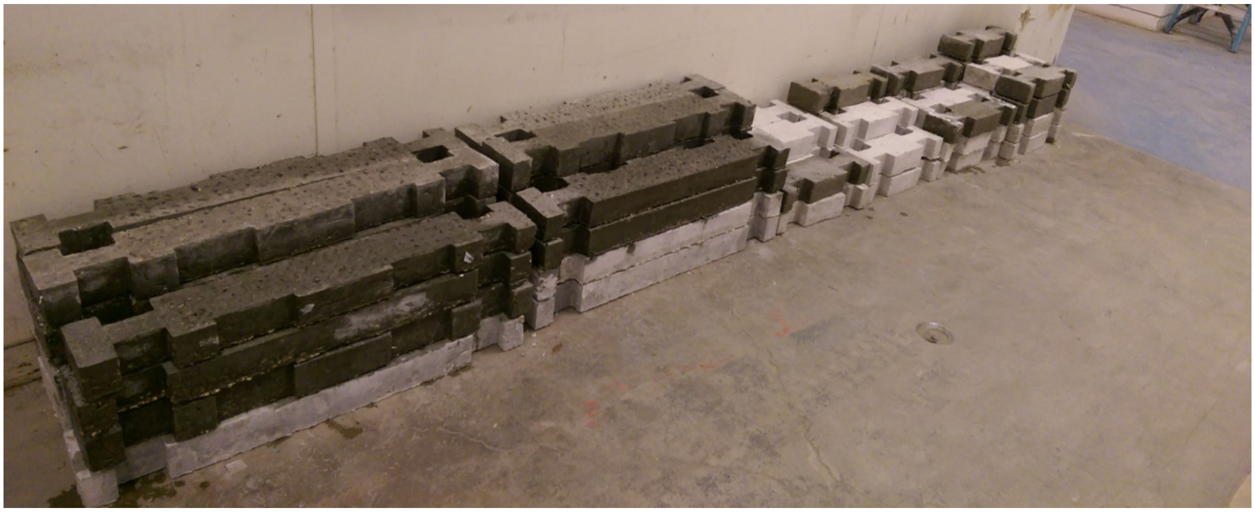

Figure S1. Large and small concrete blocks waiting for deployment.

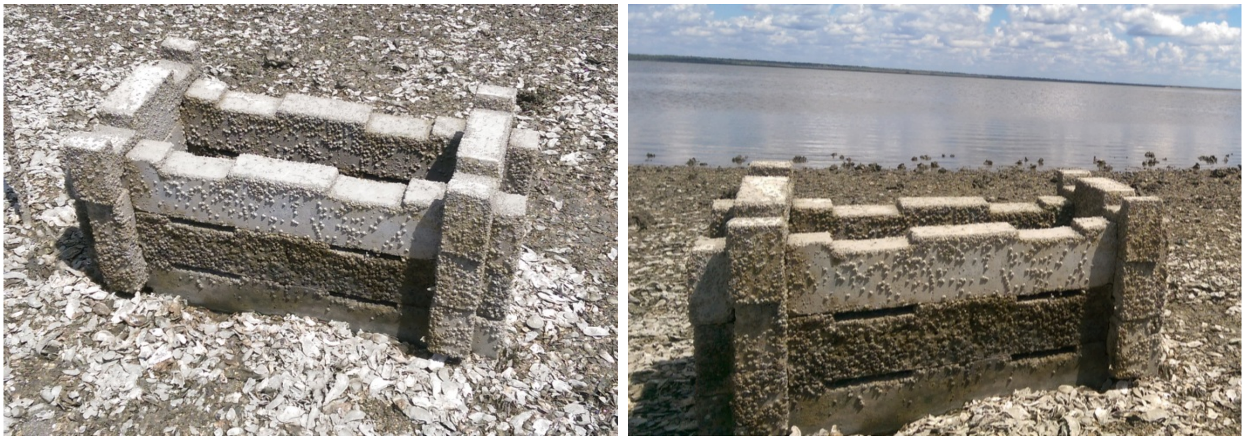

Figure S2. Deployed concrete structure.

Concrete block production procedure

The concrete structures were cast in batches using four custom-made mold units: Two large and two small mold units each produce eight blocks. The mold units, mounted on a plywood base, are composed of runners (with attached notch pieces), side plates, and braces (Fig. S3 & S4). After each batch cured for at least 24 hours, concrete blocks were demolded, and molds were reused to produce the next batch.

The production procedure is detailed below:
1. Prep the molds.
2. Batch and pour concrete.
3. Demold.

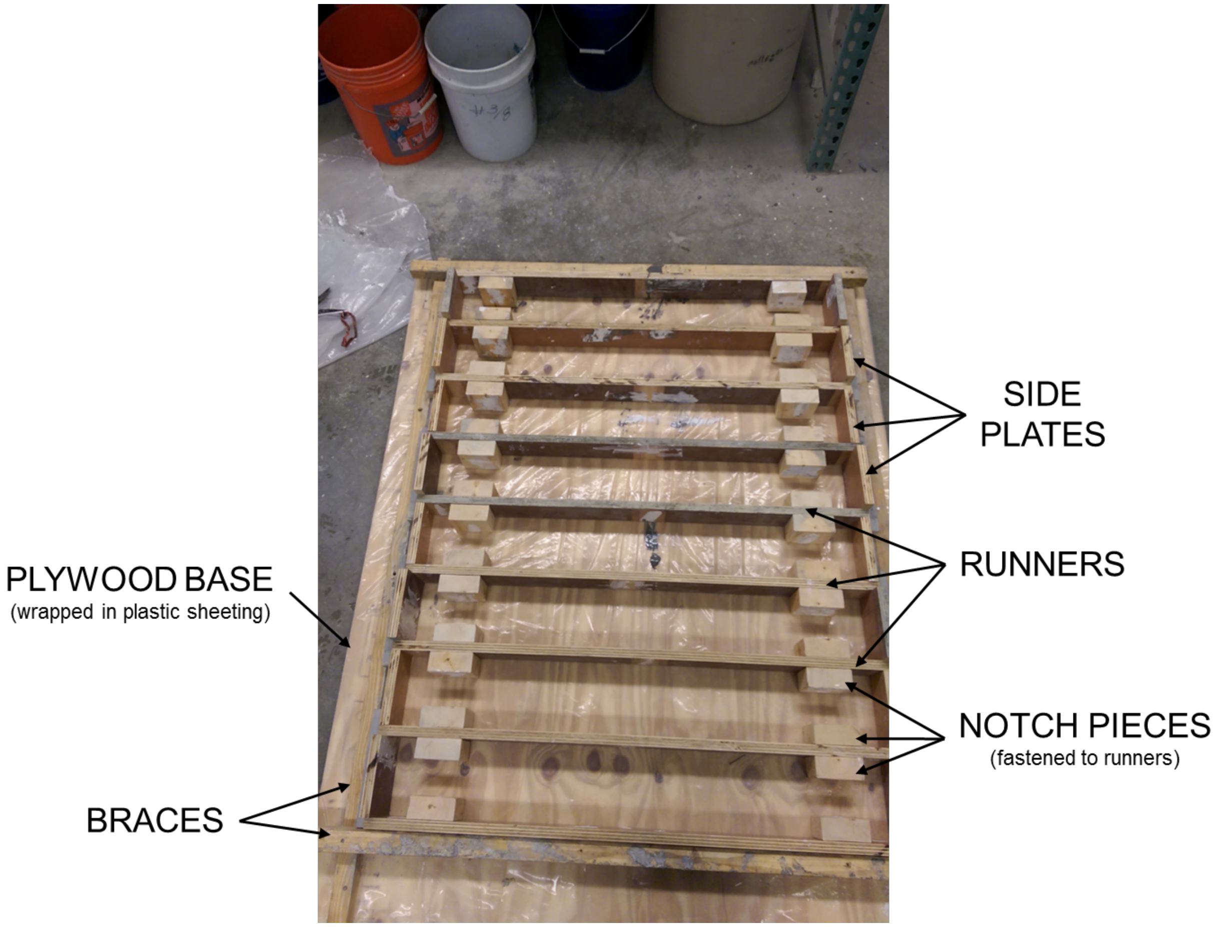

Figure S3.One of two large mold units. The units are designed for easy reconstruction and reuse after demolding each batch.

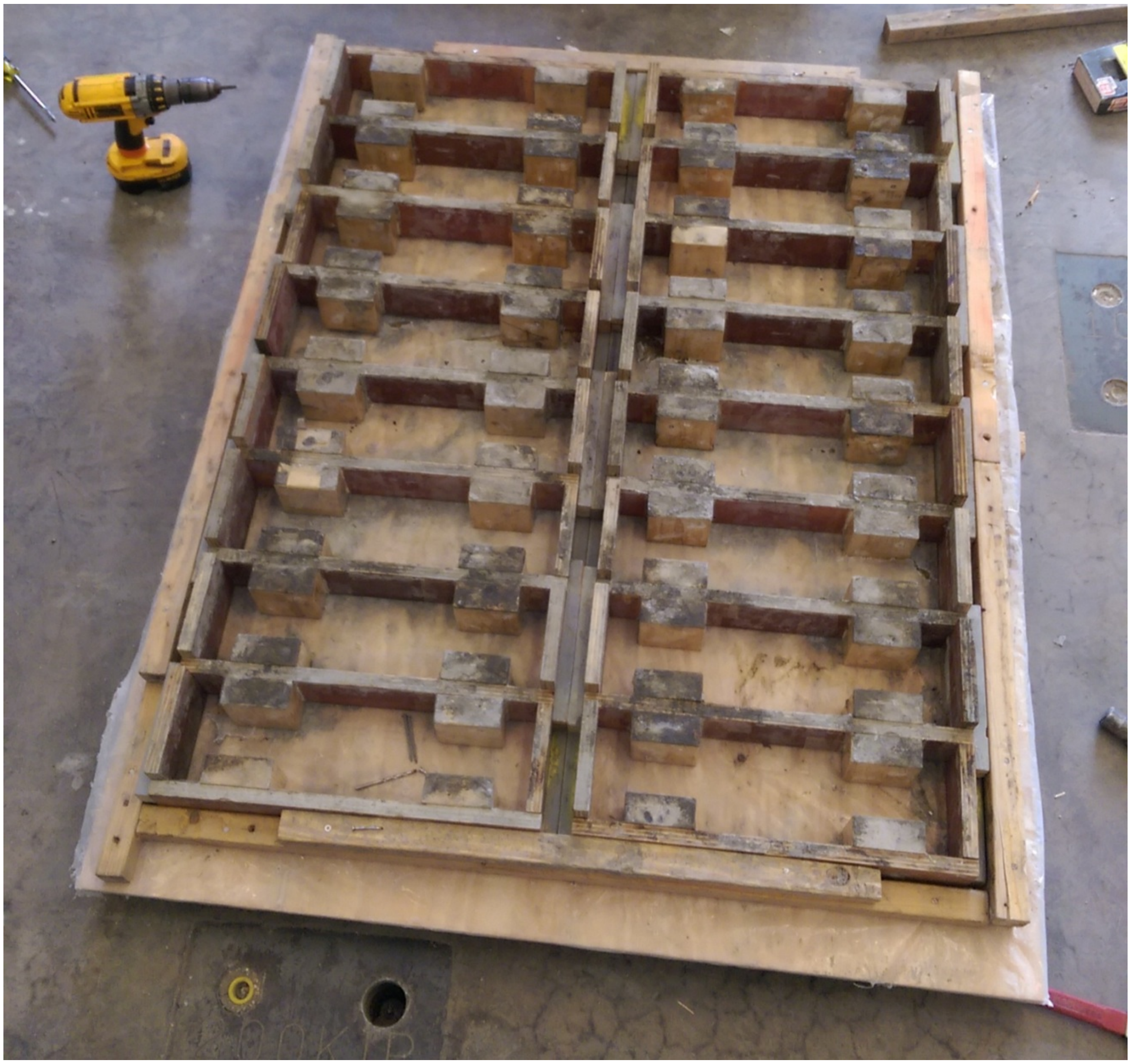

Figure S4. Two mold units for the smaller blocks fit on a single plywood base.

1. Prep the molds

1a. Replace any notch pieces knocked loose from runners during demolding. When replacing a notch piece, take care to position it correctly. The notch piece should be flush with the runner's bottom edge (check the other notch pieces to see which side is the most flush), and it should line up with the notch on the opposite side of the runner. The correct position for a notch piece is 3.8 cm (1.5 in) from the runner's edge for small molds and 6.4 cm (2.5 in) from the runner's edge for large molds. When replacing a notch piece the existing screw hole can often be used, but if the hole is too worn to make a tight fit, remove the screw and make a new hole (use a drill to make a pilot hole before driving the screw). Clamping the notch piece and the runner is recommended to ensure tight fastening.

1b. Replace tape on notch pieces. The rough texture of the wooden notch pieces grips the concrete during curing, making demolding difficult or impossible. Therefore all notches should be covered with clear packaging tape before pouring concrete (Fig. S5). First, remove any substantial bits of concrete residue and ripped or damaged tape. Then reapply tape to any exposed surfaces. The best way to apply tape to a notch piece is to stretch the tape over the notch's main surface, fold it over each side, and use a scraper with a flat edge to press the tape into the corner where the notch piece meets the runner. Then spread the excess tape along the runner, and avoid bubbles as much as possible.

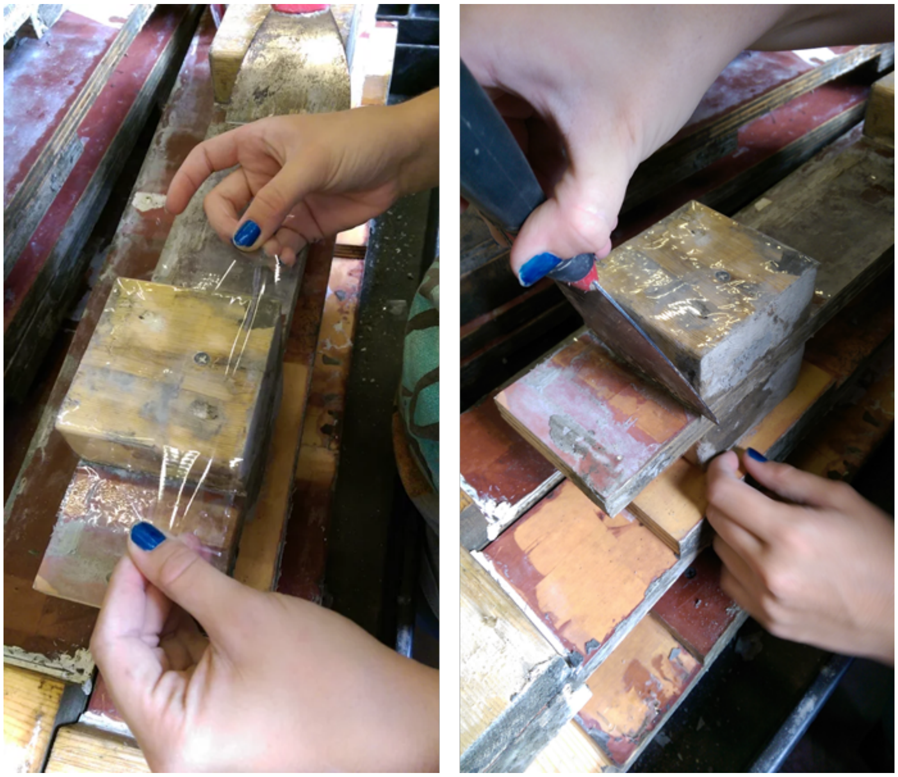

Figure S5. Apply clear packaging tape to each notch piece.

1c. Reconstruct two mold units. Each of the four mold units produces eight blocks, and there are two mold units for each type of block (large and small). There are 16 pairs of side plates—enough for two mold units—allowing 16 blocks to be produced in a single batch. For example, a

single batch can yield 16 large blocks or 16 small blocks. Side plates marked with yellow paint should be placed in the corner positions of each mold unit.

If necessary, replace the plastic sheeting on the plywood base. (The sheeting prevents the concrete from gripping the base during curing, in order to facilitate demolding.) Lay the runners out on the base. Each runner and its attached notch pieces are flush on only one side, so take care to place runners with the flush sides down against the base. Starting at one end, position the side plates between each pair of runners, starting with a pair of side plates marked with yellow paint (painted side faces up). The bars fastened to the side plates fit snugly against the runners' edges, and the plates should be placed so that the bars alternate between high and low positions (Fig. S6). Place marked side plates in the final (corner) position of the mold unit. One pair of marked side plates in each mold unit has shortened bars to accommodate the outer braces (Fig. S7).

Finally, nail the braces down around the perimeter of the mold structure. After the first brace is in place, apply force when attaching subsequent braces to ensure a tight fit. Also, after attaching each brace, examine the runners and side plates to ensure proper placement and a tight fit. Do a final check on side plates before pouring. Make sure each plate is flush against the base and standing up straight. They tend to shift easily.

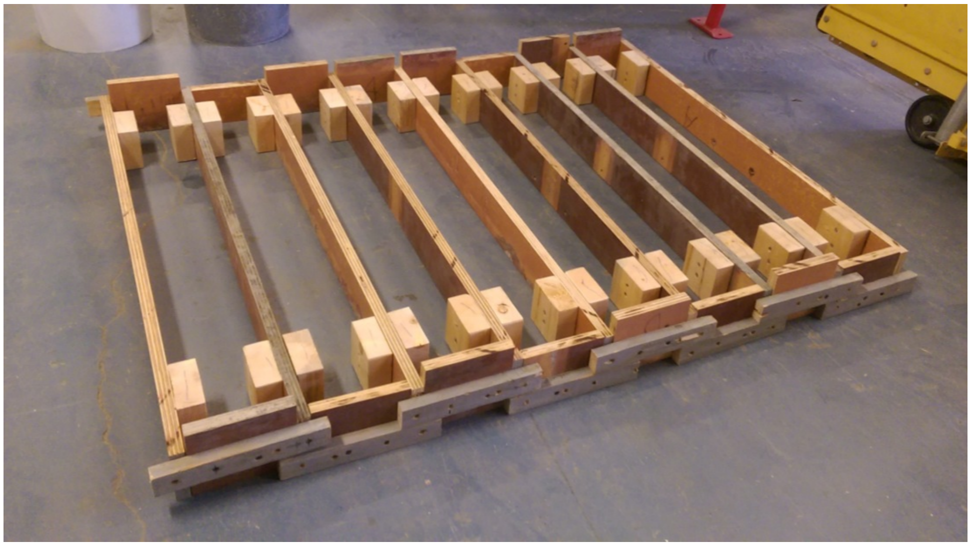

Figure S6. Place side plates so that the attached bars alternate high and low positions. Not pictured: plywood base and braces.

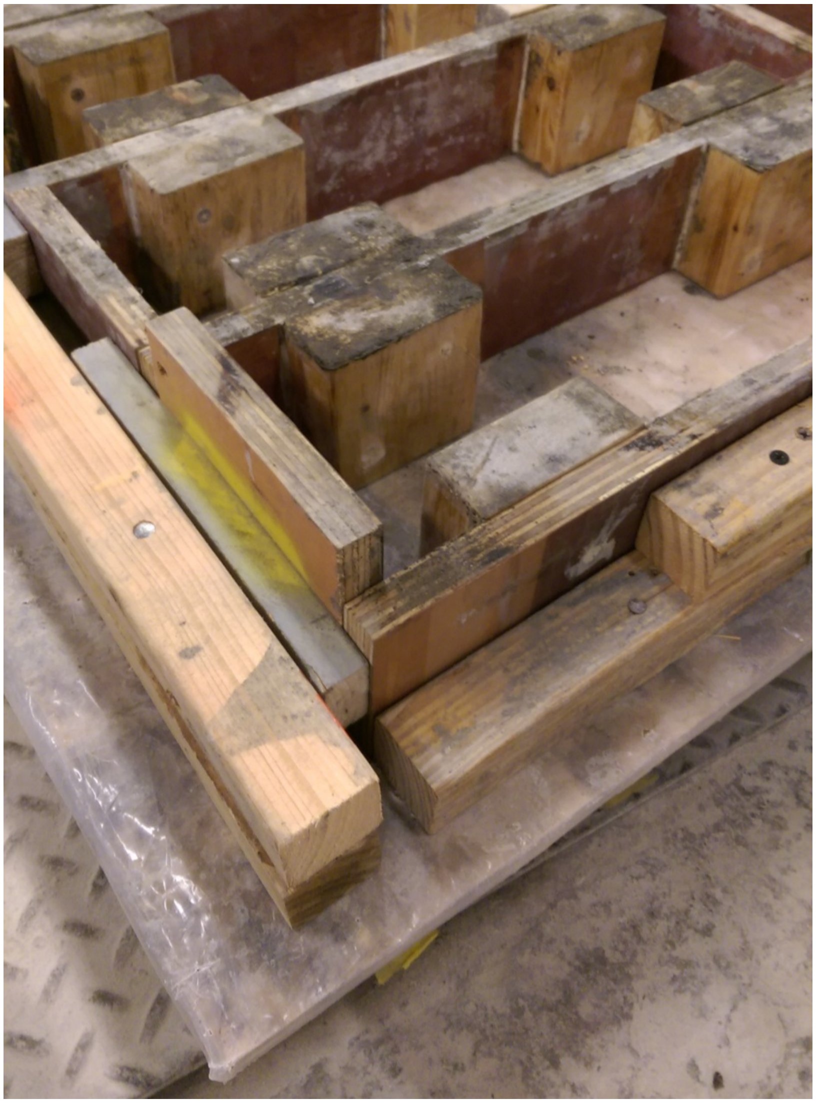

Figure S7. The corner of a small mold unit ready for pouring. Note the yellow paint indicating the orientation of the end side plate.

1d. Optional: Lubricate the mold structure with vegetable oil. The internal faces of the mold structure (those that come into contact with the concrete) can be lubricated with vegetable oil to facilitate demolding. Apply the oil with a brush immediately before pouring concrete.

2. Batch and pour concrete

2a. Batch fine aggregate, coarse aggregate, cement, water, and admixtures (PVC fibers and water reducer) (Fig. S8). Mix concrete. (See mix design below).

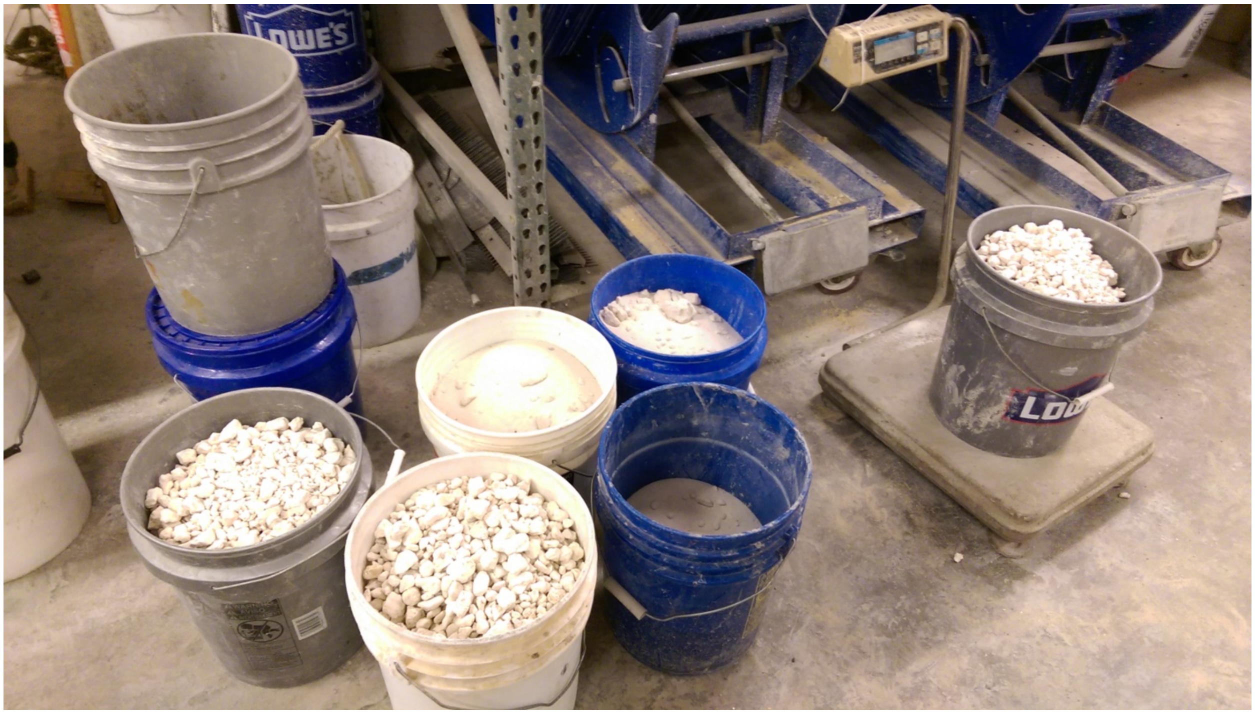

Figure S8. Batched course aggregate, fine aggregate and cement.

2b. Pour concrete from the wheelbarrow and spread it by hand into the mold structure. In the corners around notch pieces, it is best to apply the concrete in two or three layers at a time, pressing each layer into place with your knuckles, in order to fill the corners.

2c. Apply the vibrator on each of the notches for 5 to 10 seconds to remove small air bubbles (Fig. S9). Be sure to wear thick (thermal) gloves. Grip the vibrator by the black cable, not the metal, as much as possible. The vibrator can be difficult to control, so it helps to have someone hold a scrap 2”x4” or 2”x2” piece against the notch piece where the vibrator is applied in order to prevent the vibrator from sliding into the concrete. Apply the vibrator along the braces as well.

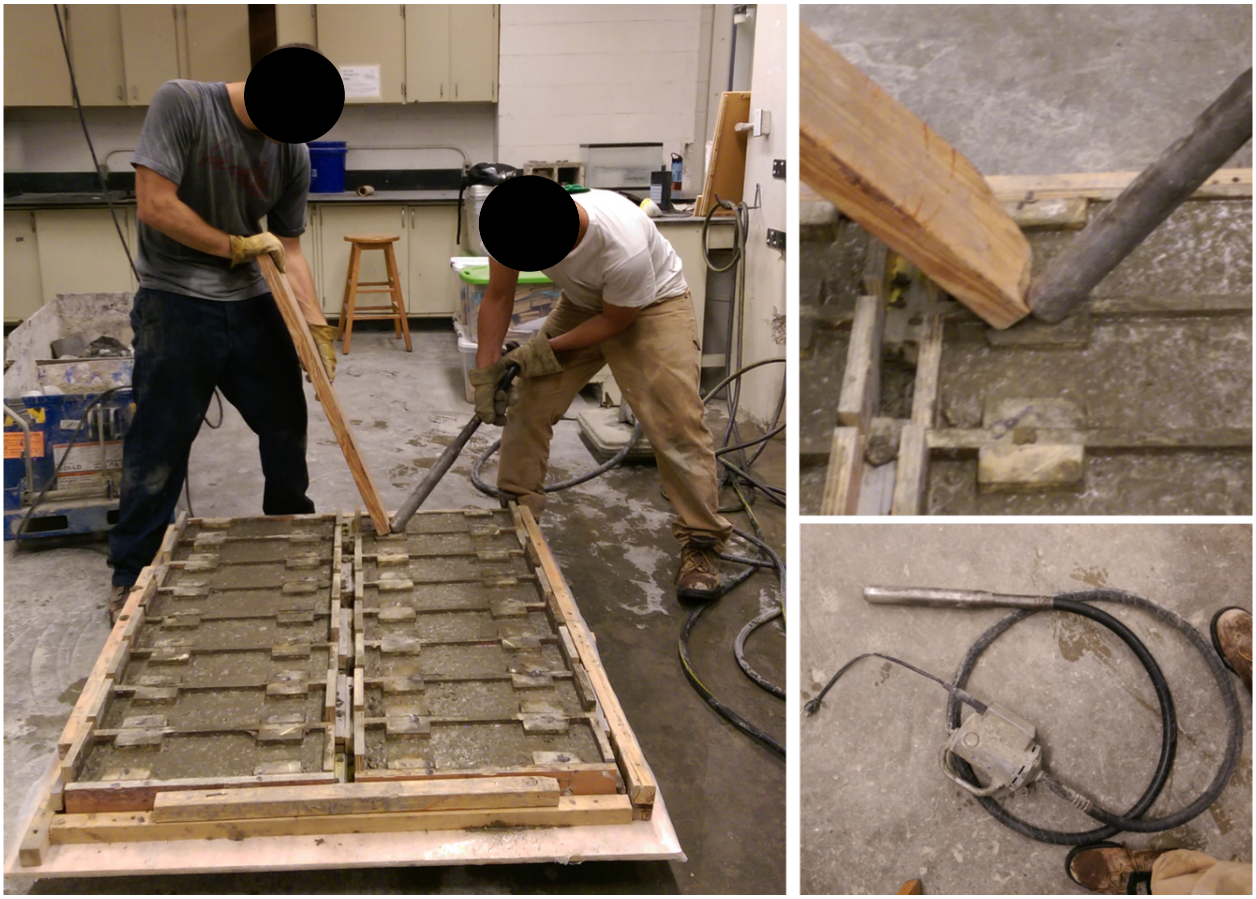

Figure S9. After pouring, apply the vibrator to eliminate bubbles. A piece of scrap can be used to keep the vibrator from sliding into the concrete.

2d. After vibrating, do a final check on side plates. Make sure they are flush against the base and standing up straight (Fig. S10). Use a piece of scrap to hammer them down if necessary. Check for any concrete leaking out of the mold.

2e. Cover the poured molds with plastic sheeting to retain moisture during curing. Spritz the surface with water once or twice during the next 4 to 8 hours.

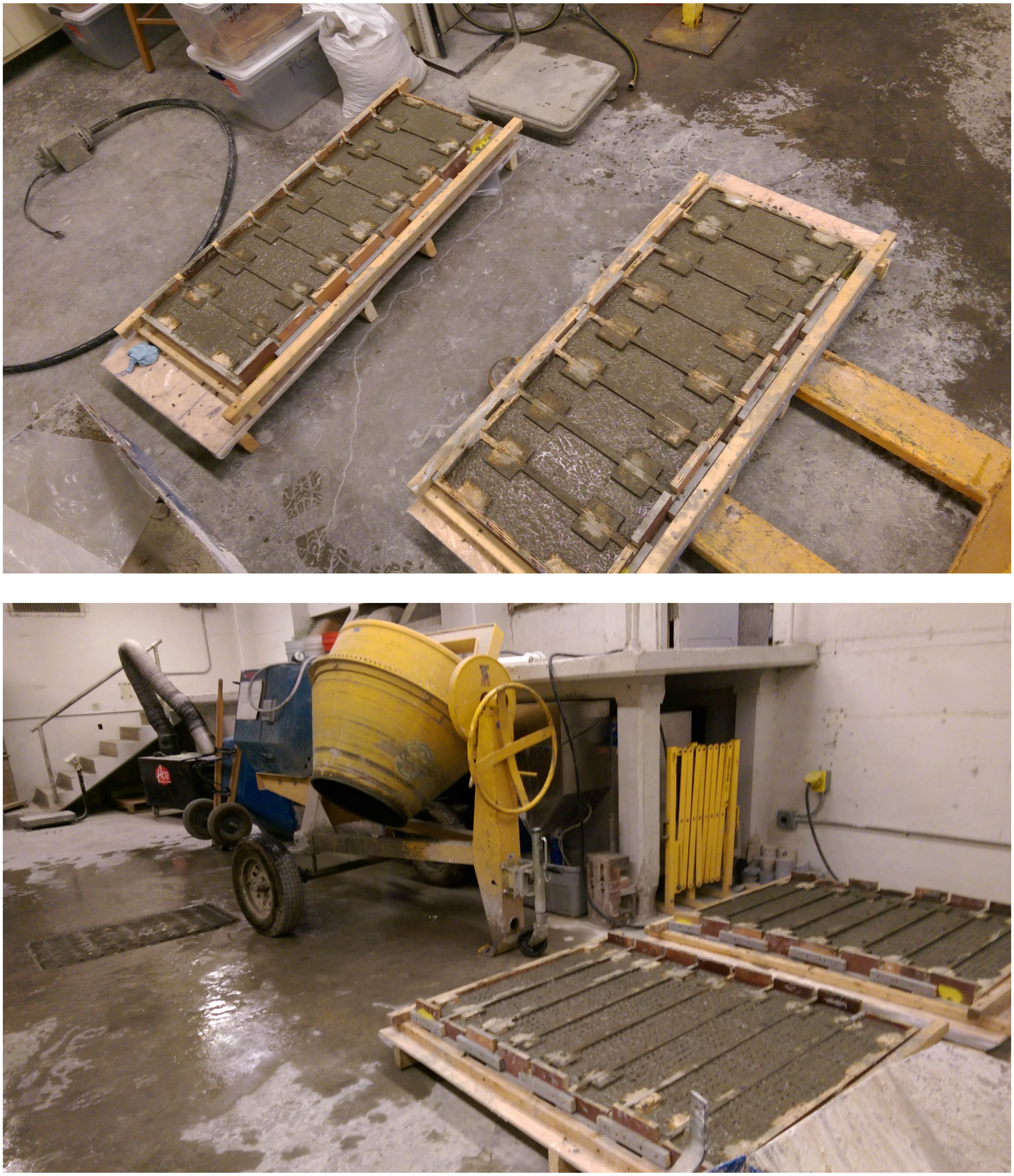

Figure S10. Poured concrete.

3. Demold

3a. After the concrete has cured for 24 to 48 hours, remove the outer braces.

3b. Starting at one end, use a rubber mallet to carefully remove runners and side plates from the blocks. A chisel may be necessary to start, and the notches are often the best place to apply force with the mallet. A hammer can also be used, but take care not to damage the mold structures (use padding).

3c. Place blocks in a hydroxide bath for days or weeks to facilitate hardening (Fig. S11).

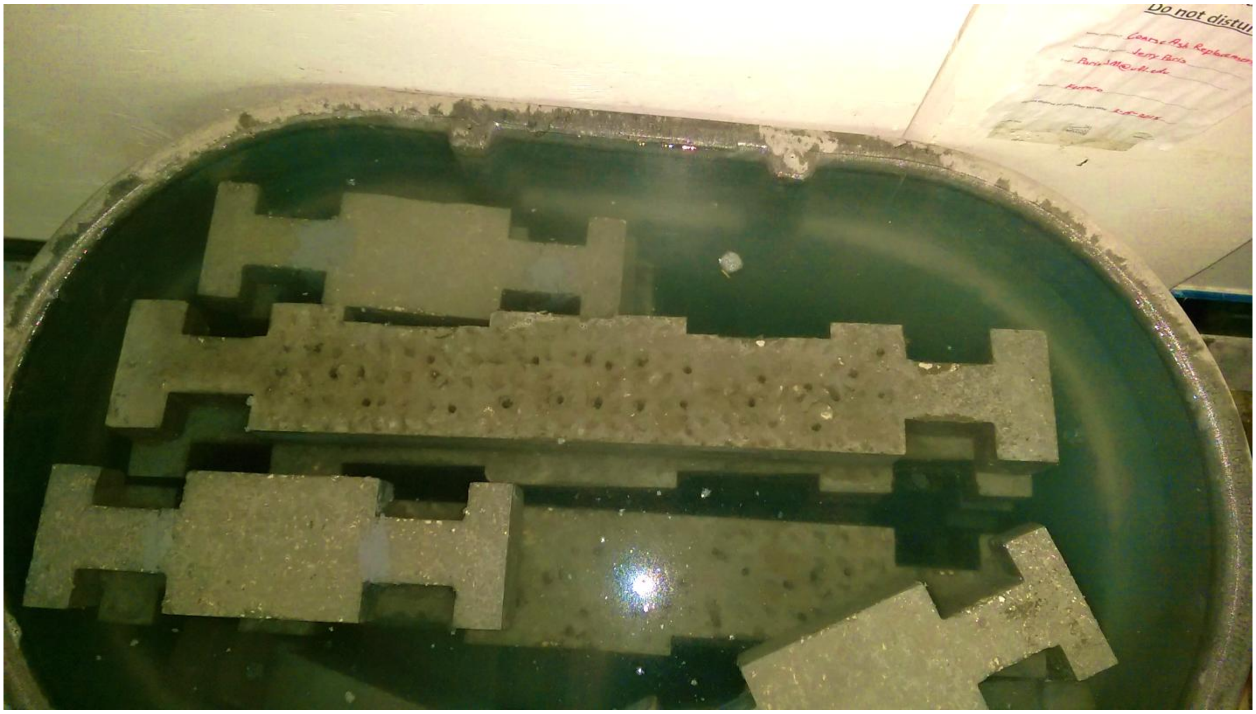

Figure S11. Hydroxide bath.

Concrete mix design

Unit wt Cement: 194.56 pcf
Unit wt Water: 62.4 pcf
Unit wt #89: 146.64 pcf
Unit wt Fine: 159.7 pcf

#89 Miami Oolite Limestone
-Absorption: 4.5%
Florida Goldhead Sand
-Absorption: 1.0%

PVC Fibers: 1.5 lb/cy
Water Reducer WRDA 60: 10oz/100lb cement
Mixer: ~7cf Capacity. Recommend doing no greater than 4cf

**Original Materials Lab Mix**	Weight (lbs)
Cement	40
Water	20
Coarse (#89 Miami Oolite limestone)	100
Fine (Florida Goldhead)	60
Water Reducer	N/A
Fibers	N/A
Yield	N/A

**Small Mix #1**	Weight (lbs)
Cement	80
Water	45
Coarse (#89 Miami Oolite limestone)	200
Fine (Florida Goldhead)	120
Water Reducer	N/A
Fibers	2.7oz
Yield	~3.1cf with 0.3cf extra

**Large Mix #1**	Weight (lbs)
Cement	160
Water	80
Coarse (large)	385
Fine	255
Water Reducer	18oz
Fibers	5.5oz
Yield	~6.2cf with 0.5cf extra
